# Mobile Health (*mHealth*) Technology for Early Detection of Psychosocial Distress in Cancer Patients: *A Scoping Review*

**DOI:** 10.2147/CMAR.S580596

**Published:** 2026-05-18

**Authors:** Taty Hernawaty, Suryani Suryani, Ida Maryati, Raini Diah Susanti, Adinda Yulia Maharani, Fillah Azzahra Syafaat, Nissa Fauziyah Sundari, Tetalia Salwa Aisyah, Silvy Erlindasari

**Affiliations:** 1 Department of Community and Mental Health Nursing, Faculty of Nursing, Universitas Padjadjaran, Bandung, West Java, Indonesia; 2 Department of Maternity and Pediatrics Nursing, Faculty of Nursing, Universitas Padjadjaran, Bandung, West Java, Indonesia; 3 Faculty of Nursing, Universitas Padjadjaran, Bandung, West Java, Indonesia

**Keywords:** *mHealth*, oncology, cancer patients, psychosocial early detection

## Abstract

**Background:**

Psychosocial screening is the systematic identification of psychological and social problems in cancer patients, including emotional distress, anxiety, and depression. *Mobile health* (*mHealth*) technology has the potential to assist in the continuous screening and monitoring of patients’ psychosocial conditions. However, evidence of its effectiveness, integration, and feasibility remains varied.

**Objective:**

To map the literature on the use of *mHealth* for psychosocial screening of cancer patients and to identify its potential, challenges, and future directions in nursing practice.

**Methods:**

A scoping review was conducted using three databases: PubMed, Scopus, and ScienceDirect.

**Results:**

A total of seven studies (n=7, including feasibility, protocol, and qualitative co-design studies) were included. Findings indicate that *mHealth* is feasible and acceptable for identifying emotional distress, monitoring psychosocial conditions, and supporting patient–provider communication.

**Conclusion:**

*mHealth* shows promising feasibility and offers potential to enhance nursing triage, referral, and patient-centered psychosocial care, and potential utility for psychosocial screening in cancer patients. However, further research is needed on long-term implementation, tool validation, data security, and integration into healthcare systems, particularly in Indonesia.

## Video Abstract

**Figure:**
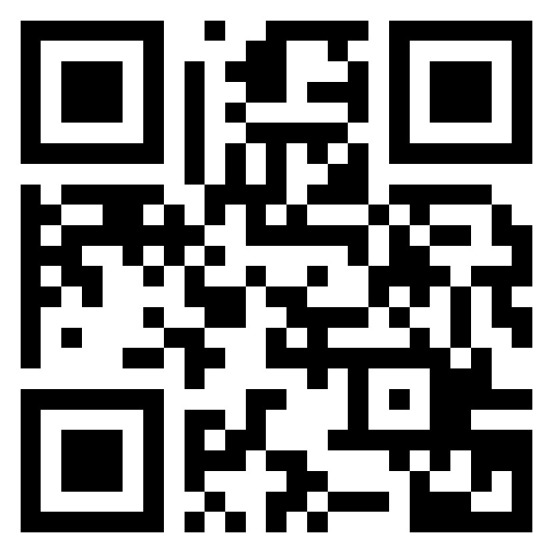

Point your SmartPhone at the code above. If you have a QR code reader the video abstract will appear. Or use: https://youtu.be/i8TbNv9S-yE

## Background

Cancer is one of the leading causes of morbidity and mortality worldwide, with a consistently increasing trend each year. According to the 2022 Global Cancer Observatory (GLOBOCAN) report, there were more than 19.3 million new cancer cases globally, with approximately 10 million deaths attributed to the disease. In Indonesia, cancer incidence shows a similar pattern, with an estimated 347,000 new cases in 2022, an increase compared with reports from previous years.^1^ This rise is attributed not only to biological determinants but also to demographic transitions, lifestyle changes, increased life expectancy, and delayed early detection,^2^ reinforcing cancer as a multidimensional public health challenge.

Beyond its significant physical burden, cancer also carries complex psychosocial consequences for patients and their families. The diagnostic process and long-term therapy often trigger emotional distress, changes in social roles, and spiritual disruptions that affect patients’ quality of life.^3^ Anxiety, depression, and feelings of helplessness have been consistently reported across oncology populations, while social withdrawal, stigma, and increased dependence on family support are common during treatment trajectories.^4^ These findings underscore that contemporary oncology care must extend beyond disease control to incorporate systematic psychosocial screening and intervention.

Psychosocial problems among cancer patients are diverse, but commonly reported issues include anxiety, depression, social isolation, uncertainty about the future, and emotional burdens on families. Depression has been reported in up to 20–25% of cancer patients, while anxiety is found in nearly one-third of patients undergoing chemotherapy.^5^ In addition, those receiving long-term treatment often struggle to adapt to social and financial changes, which can reduce treatment adherence and overall quality of life. Families, as the primary support system, also experience similar pressures, including stress related to caregiving burdens and uncertainty about prognosis.^2^ Therefore, screening and early detection of psychosocial problems are essential components of modern oncology practice.

In response, distress screening has been recommended as a standard component of comprehensive cancer care. The National Comprehensive Cancer Network (NCCN) recommends the use of the Distress Thermometer as a rapid screening tool with established threshold scores that trigger further assessment. Standardized instruments such as the Patient Health Questionnaire-4 (PHQ-4) and the Patient-Reported Outcomes Measurement Information System (PROMIS) are also widely used to assess depression, anxiety, and health-related quality of life through validated scoring systems. Unlike exploratory face-to-face clinical interviews, standardized screening tools generate quantifiable and reproducible data that can be integrated into electronic health records. This score-based approach enables the development of triage algorithms that trigger automated alerts, psychosocial referrals, and structured follow-up processes. However, the effectiveness of screening depends not only on instrument validity but also on how results are integrated into clinical workflows, including nursing triage, documentation systems, and multidisciplinary coordination.

The development of mobile health (*mHealth*) expands the potential for psychosocial screening through repeated, real-time digital self-reporting. A study by Wong et al^6^ shows that the integration of electronic questionnaire systems can improve the identification of supportive needs, while Chow et al^4^ report that text message-based screening is capable of dynamically detecting fluctuations in distress. ePRO platforms such as MyPal, evaluated by Scarfò et al^7^ and Meyerheim et al^8^ enable continuous monitoring in the context of palliative care, opening up opportunities for faster clinical response However, most studies still focus on user feasibility and acceptance, while systemic implications for nursing triage processes, psychosocial referrals, and clinical decision-making have not been comprehensively synthesized.

Several previous reviews have evaluated the effectiveness of ePRO in oncology and of digital psychological interventions in general. However, no scoping review has systematically mapped the use of *mHealth* as an initial psychosocial screening tool across cancer care settings. Specifically, no synthesis has identified the psychosocial domains covered, the implementation models within clinical workflows, or the mechanisms for using screening results to inform triage and referral decisions based on scores. Additionally, evidence regarding the long-term impact of *mHealth* use on patients’ quality of life and psychological resilience is fragmented and lacks integration. Therefore, this scoping review aims to map the characteristics, domains, and implementation strategies of *mHealth* use in the psychosocial screening of cancer patients, as well as its clinical implications, to strengthen the basis for evidence-based nursing and oncology care decision-making.

## Methods

This study employed a scoping review methodology guided by the framework developed by Arksey and O’Malley and further refined by Tricco et al^9^ through the PRISMA Extension for Scoping Reviews (PRISMA-ScR), as well as the Joanna Briggs Institute (JBI) guidelines.^10^ A scoping review was selected to systematically map the available evidence regarding the use of mobile health (*mHealth*) technology for psychosocial screening in cancer patients, identify key psychosocial domains assessed, and examine the feasibility and implementation of *mHealth* in oncology care. The review was guided by the research question:

How is mobile health (*mHealth*) technology used for psychosocial early detection in cancer patients, and what evidence exists regarding its feasibility and implementation?

The review process followed five stages: (1) identifying the research question, (2) identifying relevant studies, (3) selecting eligible studies, (4) charting the data, and (5) collating, summarizing, and reporting the results.

### Literature Search Sources

A comprehensive literature search was conducted in three electronic databases: PubMed, Scopus, and ScienceDirect. These databases were selected due to their broad coverage of oncology, digital health, nursing, psychosocial research, and provide relevant full-text articles.

### Use of Keywords

The search strategy was developed using the Population–Concept–Context (PCC) framework recommended by the Joanna Briggs Institute, where the population was cancer patients or oncology patients, the concept was mobile health (*mHealth*), mobile applications, or telehealth, and the context was psychosocial screening or psychosocial assessment. The search used the following keywords and Boolean operators: (“Cancer patients” *OR* “Oncology patients”*) AND (*“*mHealth*” *OR* “Mobile Health” *OR* “Mobile Apps” *OR* “Telehealth”*) AND (*“Psychosocial distress” *OR* “Psychosocial assessment” *OR* “Distress screening”). Search terms were adapted as appropriate for each database.

### Eligibility Criteria

Studies were included if they were published in English or Indonesian, were original research articles (including quantitative, qualitative, mixed-methods, feasibility, pilot, or protocol studies), focused on the use or development of *mHealth* technologies for psychosocial screening or assessment in cancer patients, and were available in full-text format. Studies were excluded if they did not involve cancer patients, did not include psychosocial screening or assessment, did not utilize *mHealth* technology, or were review articles, editorials, commentaries, or conference abstracts.

### Study Selection Process

**Figure 1:**
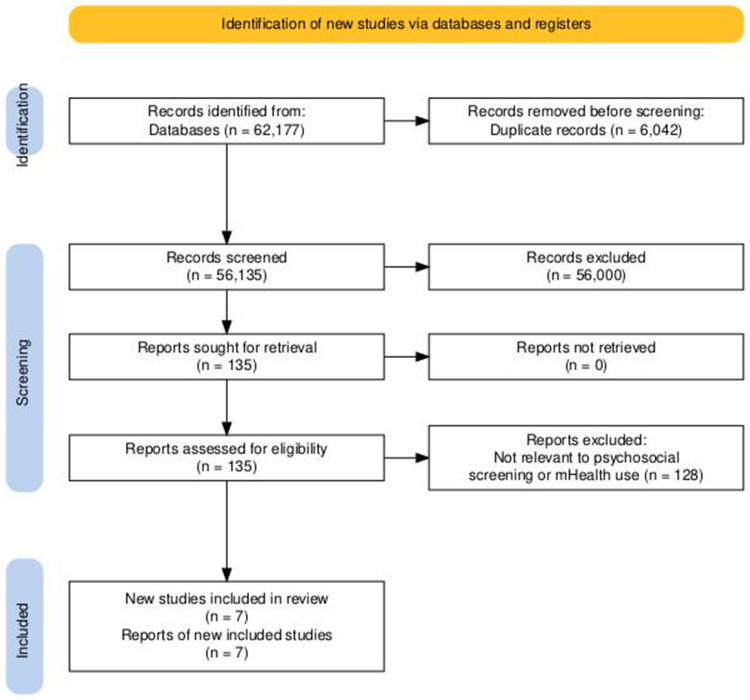
Literature Sorting Process Flowchart.

The study selection process is presented in Figure 1. The initial database search identified 62,177 records. After removing duplicate records (n = 6,042), a total of 56,135 records remained and were screened based on titles and abstracts. During this screening phase, 56,000 records were excluded because they did not meet the inclusion criteria, such as not focusing on psychosocial screening, not involving *mHealth* technology, or not targeting cancer patients. A total of 135 full-text articles were retrieved and assessed for eligibility. After full-text review, 128 articles were excluded because they were not directly relevant to psychosocial screening or the use of *mHealth* in cancer patients, or did not meet the eligibility criteria. Ultimately, seven studies met all inclusion criteria and were included in this scoping review.

### Data Charting and Data Synthesis

Data from the included studies were extracted using a standardized data charting form, which included information on author and year of publication, country of study, study design, population characteristics, type of *mHealth* technology used, psychosocial domains assessed, and key findings related to feasibility and implementation. Due to the heterogeneity of study designs and outcomes, the findings were synthesized descriptively to map the types of *mHealth* technologies used, psychosocial domains assessed, and implementation characteristics in oncology care.

## Results

This scoping review analyzes seven research articles examining the use of mobile health (*mHealth*) technology for psychosocial screening or assessment in cancer patients. The articles originate from countries such as the United States, Canada, France, Italy, Greece, Sweden, the Czech Republic, and Germany, and were published between 2018 and 2025. The studies were conducted in various cancer care settings, including academic hospitals, oncology centers, pediatric hospitals, outpatient facilities, and palliative care units. Two of the included studies were conducted within specialized psycho-oncology or cancer rehabilitation-oriented settings.

The populations included across these articles consist of cancer patients with diverse diagnoses and age ranges, including breast cancer, hematologic malignancies, and cancer in children and adolescents. Some studies focused on adult cancer patients undergoing active treatment, while others involved cancer survivors who had completed primary therapy, including childhood cancer survivors who were adolescents or young adults at the time of study. In addition, only one study employed a formal co-design approach that directly involved oncology healthcare professionals alongside cancer survivors in developing *mHealth*-based psychosocial support components. This variation demonstrates that *mHealth* applications can be adapted to different stages of the cancer trajectory—during active treatment, palliative care, and survivorship—to detect and monitor psychosocial problems.

The psychosocial problems identified in the analyzed articles encompass various aspects related to emotional distress, psychological well-being, and social support among cancer patients. Four of the seven included studies reported elevated levels of emotional distress and anxiety among adult cancer patients undergoing active treatment, highlighting the ongoing need for structured psychosocial screening and timely psychosocial support throughout the treatment trajectory. Other studies found challenges in communication between patients and healthcare providers, particularly in expressing psychological symptoms or personal care needs. Additional findings point to changes in intimacy and sexual health among breast cancer survivors, influencing interpersonal relationships and self-image. Among childhood cancer survivors, psychosocial challenges included limited social support, feelings of isolation, and difficulties adjusting after treatment, while in children and adolescents with cancer, anxiety and emotional distress were identified through both self-reporting and family observations. These findings indicate that mobile health technology has strong potential as an effective tool for identifying, monitoring, and addressing psychosocial issues across different age groups and treatment stages.

Each study in this scoping review utilized *mHealth* platforms or digital systems based on electronic patient-reported outcomes (ePRO) to conduct psychosocial screening or monitoring. Some studies used purpose-built systems, such as the MyPal ePRO system for patients with hematologic cancers and pediatric cancer patients, as well as mobile distress screening tools designed to detect real-time emotional distress in adult cancer patients. Other studies used digital platforms based on online surveys to evaluate psychological well-being, interpersonal relationships, and self-image, such as the SHINE intervention for breast cancer survivors.

Three of the included studies integrated psychosocial screening into broader digital symptom monitoring systems or proactive supportive care pathways, allowing healthcare providers to identify emotional distress and psychosocial needs in a more structured and timely manner. In co-design studies—such as those involving childhood cancer survivors—patients and healthcare professionals were directly involved in designing psychosocial screening features to suit their emotional and social needs. These findings illustrate a shift in *mHealth*-based psychosocial screening from reliance on standard questionnaires toward adaptive, interactive, and clinically integrated digital tools.

Most studies showed that the *mHealth* platforms and ePRO systems used for psychosocial screening feature simple interfaces that are easily accessible and suitable for self-completion. These systems are designed to allow patients to independently report their emotional, social, and psychosocial needs without direct supervision from healthcare providers. The findings consistently indicate high levels of acceptability, feasibility, and reliability in identifying emotional distress and psychological support needs among cancer patients.

Four of the included studies described the implementation or planned integration of *mHealth* or ePRO-based systems within clinical oncology and palliative care settings. These systems were used to facilitate structured psychosocial screening and follow-up across different phases of the cancer care continuum, including diagnosis, active treatment, and survivorship. Detailed findings on study locations, population characteristics, psychosocial aspects measured, and types of *mHealth* technologies used are presented in Table 1.

**Table 1:** Characteristics of Included Studies

Author (Year)	Design	Population	*mHealth* Type	Psychosocial Measures	Implementation Outcomes
Wong et al^6^ (2018)	Observational pilot study	428 adult breast cancer patients	Electronic Health Questionnaire System (HQS)	PROMIS; NCCN-adapted distress screening	565% referral trigger; 26.8% completed follow-up; feasible integration into intake workflow
Franzoi et al^2^ (2024)	Prospective cohort study (protocol)	Adult oncology patients	Integrated ePRO supportive care pathway	Emotional distress; psychosocial needs; symptom monitoring	Highlighted workflow integration and clinician engagement challenges
Hou et al^3^ (2025)	Qualitative co-design study	22 young adult survivors + 7 HCPs	Co-designed survivorship *mHealth* platform	Social support; engagement; personalization needs	Identified priority components; stakeholder acceptability
Shaffer et al^5^ (2024)	Randomized factorial trial study (protocol)	320 breast cancer survivors	Web-based SHINE intervention	Sexual distress; intimacy; communication self-efficacy; mental well-being	Digital module delivery; protocol phase
Scarfò et al^7^ (2021)	Randomized clinical trial study (protocol)	Adults with hematologic malignancies	MyPal ePRO platform (early palliative care)	Emotional distress; quality of life; symptom burden	Designed for real-time monitoring; usability focus
Chow et al^4^ (2019)	Longitudinal pilot study	52 adult cancer patients	SMS-based screening system	PHQ-4 (anxiety and depression)	75% compliance; <1-minute completion time; high acceptability
Meyerheim et al^8^ (2021)	Prospective feasibility protocol study	100 pediatric oncology patients	MyPal-Child gamified ePRO system	Emotional distress; quality of life	Feasibility and child engagement emphasis

## Discussion

This scoping review was conducted to synthesize findings from previous studies on the use of mobile health (*mHealth*) technology for screening psychosocial problems in cancer patients. Analysis of seven research articles indicates that *mHealth* has strong potential for early detection of psychological problems, monitoring existing psychosocial symptoms, and improving coordination of care between patients and healthcare providers. In addition to identifying potential utility and feasibility, this review also highlights how variations in intervention design, screening tools, and implementation approaches influence psychosocial screening outcomes.

The study by Wong et al^6^ showed that an electronic screening system in the form of a Health Questionnaire System containing PROMIS item banks and a distress screening tool adapted from the NCCN was able to identify patients’ psychosocial needs quickly and efficiently. Similar results were found in the study by Chow et al^4^ where *mHealth* delivered through text messages containing questions from the Patient Health Questionnaire-4 (PHQ-4) was capable of detecting fluctuations in emotional distress and was considered feasible and acceptable for use among cancer patients. These findings suggest that *mHealth*-based screening tools are not only feasible but also sensitive to short-term changes in psychosocial status, which is critical for early identification and timely intervention.

The use of electronic patient-reported outcomes (ePRO) for psychosocial screening in studies conducted by Franzoi et al^2^ and Scarfò et al^7^ demonstrated improvements in the early detection of emotional distress and changes in psychological status and symptoms through patient self-reporting via mobile devices. Monitoring through digital reporting facilitated two-way communication between patients and healthcare providers, enabling continuous monitoring, faster intervention, and improved care coordination. This reflects a shift from traditional episodic assessment toward continuous, patient-centered monitoring, which may enhance responsiveness to psychosocial needs.

Personalization and social support were also key components in the development of *mHealth* interventions. Hou et al^3^ through a co-design approach that involved young cancer survivors and healthcare professionals, identified five essential components of *mHealth* interventions: survivor connectivity, education, engagement, personalization, and support resources. These findings highlight the importance of user-centered design in improving intervention acceptability, usability, and engagement, which are essential for sustained implementation.

Two studies focused on *mHealth* interventions targeting specific psychosocial domains. The SHINE trial Shaffer et al^5^ evaluated an internet-delivered program aimed at addressing sexual distress, relationship intimacy, and communication self-efficacy among breast cancer survivors through structured digital modules. Meanwhile, the MyPal platform evaluated by Scarfò et al^7^ and Meyerheim et al^8^ emphasized patient self-reporting related to emotional distress, quality of life, and palliative care experiences. These findings suggest that *mHealth* interventions can be tailored to address specific psychosocial domains, thereby improving the precision and relevance of psychosocial screening and support.

Despite these promising findings, several important challenges and methodological limitations remain. Major implementation barriers include integration with existing clinical workflows, user adherence, and data privacy and security concerns. Furthermore, variability in study design, sample size, intervention duration, and outcome measures across the included studies limits the ability to draw definitive conclusions regarding overall effectiveness. Most studies focused primarily on feasibility and short-term outcomes, with limited evidence on long-term psychosocial outcomes, sustainability, and real-world clinical integration.

This review also has several methodological limitations. First, the small number of included studies limits the generalizability of findings. Second, as a scoping review, this study did not include formal methodological quality or risk of bias assessment, which may affect the strength of the conclusions. Third, heterogeneity in intervention types, screening instruments, and patient populations makes direct comparison across studies challenging. Nevertheless, this review provides a comprehensive overview of current evidence and identifies important gaps for future research. Further large-scale and longitudinal studies are needed to evaluate the long-term effectiveness, clinical impact, and implementation feasibility of *mHealth*-based psychosocial screening in cancer care.

## Conclusion

The findings of this scoping review suggest that mobile health (*mHealth*) technology is a feasible and acceptable approach for supporting the screening and monitoring of psychosocial problems in cancer patients. In particular, systems based on electronic patient-reported outcomes (ePRO) enable patients to report emotional distress, psychosocial needs, and well-being more efficiently, while also facilitating communication between patients and healthcare providers. These technologies may contribute to earlier identification of psychosocial concerns and support more timely and patient-centered care across different stages of the cancer trajectory.

However, the current evidence remains limited in scope and methodological strength, as the included studies were heterogeneous in design and primarily focused on feasibility, acceptability, and implementation rather than clinical effectiveness. Several challenges remain, including the need for validation and standardization of psychosocial screening instruments, variability in patient digital literacy, concerns related to data privacy and security, and integration of *mHealth* systems into routine clinical and nursing workflows.

Future research should focus on validating psychosocial screening tools delivered through *mHealth* platforms, conducting longitudinal and implementation studies to evaluate their impact on patient outcomes, and examining integration strategies in diverse healthcare settings, including resource-limited contexts. In addition, culturally appropriate adaptation, digital equity, and sustainable integration into healthcare systems should be prioritized to ensure the effective and responsible use of *mHealth* in psychosocial cancer care.
